# Calcium Absorption from Fortified Ice Cream Formulations Compared with Calcium Absorption from Milk

**DOI:** 10.1016/j.jada.2009.02.017

**Published:** 2009-05

**Authors:** Regine M. van der Hee, Silvia Miret, Marieke Slettenaar, Guus S.M.J.E. Duchateau, Anton G. Rietveld, Joy E. Wilkinson, Patricia J. Quail, Mark J. Berry, Jack R. Dainty, Birgit Teucher, Susan J. Fairweather-Tait

## Abstract

**Objective:**

Optimal bone mass in early adulthood is achieved through appropriate diet and lifestyle, thereby protecting against osteoporosis and risk of bone fracture in later life. Calcium and vitamin D are essential to build adequate bones, but calcium intakes of many population groups do not meet dietary reference values. In addition, changes in dietary patterns are exacerbating the problem, thereby emphasizing the important role of calcium-rich food products. We have designed a calcium-fortified ice cream formulation that is lower in fat than regular ice cream and could provide a useful source of additional dietary calcium. Calcium absorption from two different ice cream formulations was determined in young adults and compared with milk.

**Subjects/setting:**

Sixteen healthy volunteers (25 to 45 years of age), recruited from the general public of The Netherlands, participated in a randomized, reference-controlled, double-blind cross-over study in which two test products and milk were consumed with a light standard breakfast on three separate occasions: a standard portion of ice cream (60 g) fortified with milk minerals and containing a low level (3%) of butter fat, ice cream (60 g) fortified with milk minerals and containing a typical level (9%) of coconut oil, and reduced-fat milk (1.7% milk fat) (200 mL). Calcium absorption was measured by the dual-label stable isotope technique.

**Statistical analysis:**

Effects on calcium absorption were evaluated by analysis of variance.

**Results:**

Fractional absorption of calcium from the 3% butterfat ice cream, 9% coconut oil ice cream, and milk was 26%±8%, 28%±5%, and 31%±9%, respectively, and did not differ significantly (*P*=0.159).

**Conclusions:**

Results indicate that calcium bioavailability in the two calcium-fortified ice cream formulations used in this study is as high as milk, indicating that ice cream may be a good vehicle for delivery of calcium.

Bone health is a major public health concern in industrialized countries (1,2). The current consensus is that 1.66 million hip fractures occur each year worldwide, and the incidence is predicted to increase fourfold by 2050 because of the increasing number of older people (3). Interestingly, age-adjusted incidence rates are many times higher in affluent countries than in sub-Saharan Africa and Asia (3). Although osteoporosis is a complex condition affected by genotype, diet, and lifestyle, calcium and vitamin D are both recognized as key nutrients in promoting bone health (3).

Prevalence of dietary calcium insufficiency is not known, but there is evidence suggesting that people of all ages worldwide (4), including in the United States (5), fail to consume adequate amounts of calcium. Low calcium intakes are very common in developing countries because of the infrequent consumption of dairy products. Childhood and adolescence are critical times to optimize peak bone mass and inadequate consumption of calcium in these years increases the risk of osteoporosis and bone fractures in later life (6). Dairy products, in particular milk, are recognized as good sources of bioavailable calcium and there have been several health and nutrition campaigns focused on increasing intakes of dairy foods (7-9). However, trends in the consumption of beverages, “particularly among children and adolescents,” show that soft drinks are replacing milk, thereby affecting the intake of many essential micronutrients (10,11). It is possible that dairy products such as milk and cheese are not especially exciting or appealing and this could contribute to continued low consumption of these products, despite health campaigns. Ice cream is a widely consumed product and can provide an additional source of calcium that can easily be assimilated into the diet.

It is essential that calcium-fortified ice cream meet certain design criteria. It should not only be appealing to consumers so that it provides a substantial contribution to daily calcium intake, but should also be lower in fat content than regular ice cream, particularly when considering its consumption by children. It is also important to evaluate the bioavailability of the calcium in fortified products. In the case of ice cream, insoluble calcium soaps can form in the presence of certain fats, leading to a decrease in calcium absorption (12-14). Therefore, we designed a study to evaluate the effect of different types of fat (butterfat and coconut oil), used in the formulation of two ice cream products, on calcium absorption.

The aim of the study was to measure fractional calcium absorption from two different formulations of ice cream and to compare it with reduced-fat milk. True fractional absorption was determined using the dual-label stable isotope technique.

## Methods

### Subjects

Eighteen subjects (four men and 14 women) aged 25 to 45 years, with no medical history of disease known to influence calcium absorption or metabolism, were recruited from the general public of The Netherlands. Body weight of the subjects was measured (kg ±0.1) with a calibrated digital scale (Seca, Hamburg, Germany) wearing indoor clothing without shoes, wallet, keys, or mobile phone. Height measurements (cm) were performed along the median line of the person's back, without shoes, using a height measurement device (Seca) permanently attached to the wall. Body mass index was calculated as kg/m^2^. Single measurements were carried out by a trained nurse, followed by a physical examination, including vital signs. Urine and fasting blood samples were collected for clinical laboratory tests to ensure that they met inclusion criteria. Volunteers were fully informed of the aims and purposes of the study and gave written informed consent. The study protocol was approved by the Medical Ethics Committee of the Wageningen University, The Netherlands.

### Preparation of Stable Isotope Solutions

A dual-label stable isotope technique was used, which involves simultaneous administration of two different stable isotopes, one given orally and one intravenously. True fractional absorption of calcium was calculated from the enrichment of both isotopes in blood samples by taking into account the amounts administered and the natural abundance of the stable isotopes (15,16). The stable ^44^Ca isotope was supplied by Cambridge Isotope Laboratories (Andover, MA) as ^44^Calcium chloride (CaCl_2_) (Lot l1-8993B) with a chemical purity of 99.96% and isotopic enrichment of 96.4%. The ^44^Ca was used in the test products and taken orally. The stable ^42^Ca isotope was supplied by Cambridge Isotope Laboratories as ^42^Calcium chloride (CaCl_2_) (Lot l1-8993A), with a chemical purity of >98% and isotopic enrichment of 87% and was administered intravenously. Calcium isotope solutions (^42^Ca) for intravenous administration (5.0 mL) were prepared by the hospital pharmacy. Vials containing 5.0 mL solution (4.9 mg ^42^Ca) were stored at 4°C until use.

### Preparation of Test Products

Composition of test products is given in Table 1. Test products were 3% butterfat ice cream, 9% coconut oil ice cream, and reduced-fat milk (British Dairygate fresh pasteurized semi-skimmed milk containing 1.7% fat, British Dairygate, Lexington, UK). Calcium content of the milk was 0.11% wt/wt, ie, about 220 mg per 200-g portion. The ice cream formulations were designed to contain as much calcium in a 60-g portion as a 200-g glass of milk (220 mg calcium per 60 g or 0.37% calcium in the formulation). Quantity consumed was weighed to ensure that the calcium doses were identical on each occasion.

The ice cream mixes were prepared in a tank by adding sugar, stabilizers, emulsifiers, reduced-fat milk powder, oils, and flavors to water (80°C), and the mix was blended for 10 minutes at ∼60°C to 70°C. The mix was homogenized, pasteurized, cooled, and stored at 4°C for approximately 2 hours prior to the addition of the ^44^Ca isotope. Levels of calcium measured in the base samples before the addition of ^44^Ca isotope were 227 mg for 3% butterfat ice cream (per 60-g portion), 224 mg for 9% coconut oil ice cream (per 60-g portion), and 223 mg for reduced-fat milk (per 200-g portion).

All test products were extrinsically labeled with ^44^CaCl_2_ by adding the ^44^Ca-isotope solution to the ice cream mixes and the fresh reduced-fat milk while stirring. Test products were stored overnight at 4°C to allow for isotopic exchange between the label and the calcium naturally present in the mixes, then aliquoted into individual portions (60 g for the ice creams, 200 g for the milk) and stored frozen at −25°C. All samples were routinely tested for microbiological safety. The samples were transported frozen from the Unilever Research & Development site in Colworth, UK (site of manufacture) to the metabolic ward of TNO Zeist, The Netherlands (study site). The ice cream samples were consumed frozen at −18°C. The milk was thawed for 24 hours in a refrigerator and consumed at +4°C.

### Study Design

The study was a randomized, reference-controlled, double-blind cross-over study with three 1-day intervention periods corresponding to the three test products. The intervention days were separated by two 13-day washout periods. Participants were instructed by a trained nutritionist to follow their habitual diet and to record their dietary intake and physical activity on the days before, during, and after the intervention in a self-administered food consumption diary. In total, three 2-day diaries were collected per participant. These diaries were reviewed for completeness and dietary calcium intake was assessed on the preintervention and intervention days. Intake of nutrients and calcium were calculated according to Dutch food-composition tables (NEVO table) and randomly double-checked (17). Adverse events were registered and classified according to the *International Classification of Disease*, ninth revision.

Prestudy laboratory tests of fasting blood and urine from the volunteers were carried out to evaluate various parameters. Tests included hematology, serum clinical chemistry profile, dipstick urinalysis, and 25-hydroxyvitamin D in serum. Relevant baseline characteristics of the volunteers are shown in Table 2. All parameters were within the normal range and met inclusion criteria.

On each intervention day (days 1, 14, and 28), after an overnight fast, the volunteers were instructed to consume the test product within 5 minutes after the standardized breakfast, which consisted of two slices of white bread with margarine and strawberry jam and 125 mL water (breakfast contained 15.3 mg Ca, excluding the test product). After consumption of the test product, the volunteer rinsed the empty bottle or container with 125 mL mineral water (5.7 mg calcium) and drank this to ensure that the entire test product was consumed. Compliance was 100%. Within 30 minutes of oral administration of the test product, 5.0 mL (4.9 mg) ^42^Ca was infused intravenously during a period of 1 hour under medical supervision. Volunteers consumed only mineral water (5.7 mg Ca per 125 mL) for 4 hours following consumption of the test product and total intake was recorded. All test products contained similar quantities of the stable calcium isotope (^44^Ca) as well as similar quantities of total calcium. Blood samples were taken before and 24 hours after consumption of the test products.

### Preparation of Serum Samples

Serum aliquots of 0.5 mL were added to 1 mL 60% vol/vol HNO_3_ (Ultrapur, Merck, Darmstadt, Germany) in quartz tubes and placed in a heating block at 80°C for 1 hour. Once cooled, 0.5 mL H_2_O_2_ (Ultrapur, Merck) was added to each tube and placed in a closed ultraviolet digestion system (model 705, Metrohm LTD, Herisau, Switzerland) for 2 hours at 90°C. Clear digests were transferred into vials to dry and were then redissolved in 0.8 mL 0.1 mol/L HNO_3_ and decanted into Falcon tubes, to which 0.2 mL saturated ammonium oxalate (Sigma-Aldrich, St Louis, MO) solution (pH 8 to 9) was added. After overnight incubation at room temperature, samples were centrifuged at 3,500*g* for 10 minutes. Precipitates were washed and dried. Each pellet was dissolved in 2 mL 2% HNO_3_ and samples were further diluted to a calcium concentration of 2 ppm for inductively coupled plasma mass spectrometry analysis. Certified serum controls (Lyphochek, Assayed Chemistry Control, Level 1, Bio-Rad, Hercules, CA) and reagent blanks were processed with each set of serum samples.

### Calcium and 25-Hydroxyvitamin D Measurements

Total calcium of the test meal was determined by using a dry-ash preparation method and atomic absorption spectrometry (model 3300 Perkin Elmer, Norwalk, CT) as described previously (16). For measurements of serum 25-hydroxyvitamin D, serum was separated from whole blood and stored at −80°C before analysis. All samples were analyzed in one batch by radioimmunoassay according to the method described by Jongen and colleagues (18).

### Inductively Coupled Plasma Mass Spectrometry Analysis

Isotope ratios were measured on a single focusing multicollector mass spectrometer (Isoprobe, GV Instruments, Manchester, UK) using a desolvating sample introduction system with a microconcentric nebulizer (Aridus and T1H, Cetac, Omaha, NE). Each sample run on the inductively coupled plasma mass spectrometry was bracketed by a certified standard (NIST915) (19) and corrected for any mass bias. Samples were run in duplicate and 10% (randomly assigned) in triplicate. Baseline correction was used to ensure that (subject) natural variation was eliminated as a potential source of error. Total calcium concentration of the ice cream and milk samples were determined by the isotope dilution technique, following the addition of a known quantity of ^48^Ca to each sample prior to processing.

### Calculation of Fractional Absorption

Fractional calcium absorption is calculated from the ratio of the orally (^44^Ca) and intravenously (^42^Ca) administered stable isotopes measured in serum, expressed as the fraction of the administered dose. This technique assumes that the oral tracer, once absorbed, follows the same kinetics as the intravenous tracer and natural calcium. A matrix inversion technique (20) was used to yield the mole fractions of each calcium source present in serum samples. By correcting these for the quantity of dose given, the fractional calcium absorption can be estimated.

### Statistical Analysis

Based on evidence in the literature (21), the variance of the response variable (ratio of ^44^Ca and ^42^Ca) was estimated to be approximately 12%. Power calculations revealed that to detect a difference of 30% in the mean fractional calcium absorption (approximately 12.5 in absolute value) between the treatments, with a power of 80% and an α=.05 (two-sided), a sample size of 15 volunteers would be necessary. In order to allow for possible dropouts, we planned to recruit 18 healthy volunteers into the study.

Statistical analysis on data was performed using the SAS software (version 9.1.2, 2004, SAS Institute Inc, Cary, NC). Descriptive analyses consisting of distribution statistics (number of available observations, mean, standard deviation, and 95% confidence intervals) for continuous data are presented. Effects of treatments on calcium absorption were evaluated by analysis of variance. The model includes the treatment, period, and subjects as factors.

## Results

Sixteen participants completed the study. Two dropped out after the first intervention for medical reasons not related to the study or test products. Baseline characteristics of the participants, including serum 25-hydroxyvitamin D and mean daily intake of calcium and macronutrients are reported in Table 2. None of the participants had 25-hydroxyvitamin D concentrations indicative of vitamin D deficiency, ie, <10 ng/mL (<25 nmol/L), and all met the inclusion criteria of >20 ng/mL (>50 nmol/L). There was no significant change in the intake of nutrients or calcium during the study (*P*>0.05). Mean daily intake of calcium was 694±442 mg, calculated from the food-consumption diaries on each (pre-) intervention day (between-subject range=203 to 1,726 mg/day).

Based on triplicate analysis of baseline and enriched serum samples, the within-run precision (% coefficient of variation) of the isotope ratios was 0.15% (±0.12) for the ratio of ^42^Ca to ^40^Ca, and 0.32% (±0.27) for ^44^Ca to ^40^Ca.

Mean calcium absorption values obtained from the three test products are shown in the Figure. Calcium absorption from the 3% butterfat ice cream was 26%±8%; absorption from the 9% coconut oil ice cream was 28%±5% and absorption from reduced-fat milk was 31%±9%. No significant difference in fractional calcium absorption from the 3% butterfat ice cream, 9% coconut oil ice cream, or reduced-fat milk was observed (*P*=0.159), and there was no significant difference between the two ice cream formulations. Results were not influenced by the test meal sequence (*P*>0.05). Values for mean percentage calcium absorption from the three test products were within the range of published values for milk, namely, 15% to 44% (16,22-24).

## Discussion

Calcium and vitamin D are required for bone accretion, and there are reports that higher intakes of calcium are related to increased bone mass in children, young adults, and postmenopausal women, thereby preventing osteoporosis and risk of fracture in later life (25). In industrialized countries, the main dietary sources of calcium are milk and other dairy products, which supply 50% to 80% of dietary calcium. Changes in beverage consumption, with milk being replaced by soft drinks (10,11) can result in lower intakes of calcium, therefore, calcium-fortified foods can make a valuable contribution, particularly when the products appeal to the subgroups of the population who require additional calcium to promote bone health (26).

Ice cream is a palatable and widely enjoyed food product that could provide a useful source of calcium in the diet. It naturally contains calcium and can be additionally fortified to provide substantial amounts (200 mg) without impacting on taste and flavor. Ice creams used in this study were a low-fat dairy ice cream (3% butterfat, normal levels of sugar) enriched with milk minerals and a typical European vegetable-fat ice cream (9% coconut oil, normal levels of sugar and calories) enriched with milk minerals. Levels of milk minerals provided the same amount of calcium in a 60-g portion of ice cream as in a 200-g portion of milk.

Bioavailability of calcium in ice cream has not been measured previously. We therefore conducted the current study to compare the absorption of calcium from ice cream and milk. In order to compare fractional absorption of calcium from different food products, we performed the study in volunteers with normal vitamin D status (35.7±6.4 ng/mL [86.6±15.5 nmol/L]) and within a homogenous age group (25 to 45 years). Vitamin D levels were considered adequate, taking into account the fact that the study took place between November and December at a latitude above 35° (Amsterdam is 52°), where winter sunlight lacks sufficient ultraviolet B required to produce provitamin D_3_(27).

There were no statistically significant differences in calcium absorption from the two ice cream formulations and milk, demonstrating that calcium-fortified ice cream is an effective vehicle for delivering calcium. The two ice creams gave similar results, despite differing fat type and content (3% butterfat vs 9% coconut oil). Butterfat contains mainly palmitic acid, while coconut oil is rich in lauric acid. Koo and colleagues (28) report that regular consumption of palm oil in infant formulas reduces intestinal absorption of calcium and lowers bone mineral mass. Digestion of palm oil results in a high proportion of free palmitic acid in the intestine, and free palmitic acid and other long-chain unsaturated free fatty acids form salts with divalent cations (mainly calcium), generating fatty-acid soaps (29), which are not well-absorbed and, therefore, reduce calcium bioavailability (30,31). However, the quantity of butterfat (and consequently of palmitic acid) present in infant formulas is about 10 times higher than that present in ice cream, which presumably explains the disparity between results from studies on modified infant formulas and ice cream. Because we found no evidence of decreased calcium absorption in butterfat ice cream, we conclude that at the typical fat concentrations in ice cream, palmitic acid does not have an adverse effect on calcium absorption.

It is essential that calcium-fortified products meet certain design criteria that enable them to be readily incorporated within a normal diet. The two ice cream formulations were formulated responsibly in terms of nutritional profile, with one portion containing 220 mg calcium and either 91 kcal for the 3% butterfat formulation, or 115 kcal for the 9% coconut oil formulation. In terms of energy content, this is comparable with the nutritional value of a banana, a bowl of yogurt with muesli, or half of a chocolate chip cookie.

## Conclusions

Results from this study show that the fractional absorption of calcium from the two ice cream formulations is not statistically different from reduced-fat milk when all products contained equal amounts of calcium. In addition, no statistical difference in fractional absorption of calcium was found between the two different ice cream formulations. These findings indicate that absorption of calcium from both ice cream formulations is as good as milk and illustrate that the typical ingredients and frozen format of ice cream do not negatively influence calcium absorption. Calcium-fortified ice cream provides a useful dietary source that can contribute to total daily intake of calcium.

## Figures and Tables

**Figure:**
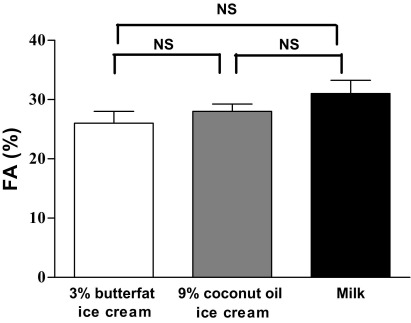
Mean percent fractional absorption (FA) of calcium from 3% butterfat ice cream (26%±8%; coefficient of variation [CV]=30%; range=15% to 39%), 9% coconut oil ice cream (28%±5%; CV=18%; range=17% to 38%) and milk (31%±8%; CV=26%; range=17% to 49%) measured in serum collected 24 hours after dosing (n=16). NS=not significant (*P*<0.05).

**Table 1:** Composition of the two different ice cream formulations used in the dual-label stable isotope calcium bioavailability study^a^

Ingredient	3% Butterfat ice cream	9% Coconut oil ice cream	Reduced-fat milk
Total calcium (mg/portion)^b^	227	224	223
^44^Calcium (mg/portion)	8.9	8.6	8.2
Energy (kcal/portion)	91	115	95
Protein (g/portion)	2.5	1.4	6.6
Fat (g/portion) (saturates)	1.9 (1.3)	5.6 (5.2)	3.3 (2.1)
Carbohydrates (g/portion) (sugars)	15.7 (13.8)	14.6 (12.8)	9.7 (9.7)
Reduced-fat milk powder (wt/wt %)	11.9	4.0	
Whey powder 30% (wt/wt %)^c^	—	3.0	
Milk minerals (wt/wt %)^d^	0.93	1.22	
Sucrose (wt/wt %)	15.2	11.5	
Low-fructose corn syrup (wt/wt %)	—	11.7	
Glucose syrup (wt/wt %)	4.8	—	
Locust bean gum (wt/wt %)	0.171	0.171	
Carrageenan L100 (wt/wt %)	0.019	0.019	
Butterfat (wt/wt %)^e^	3.0	—	
Coconut oil (wt/wt %)^f^	—	9.0	
Emulsifier (wt/wt %)	0.12	0.285	
Vanilla flavor (wt/wt %)	0.014	0.014	
Water (wt/wt %)	63.85	59.09	

a Macronutrients and calcium were expressed per portion size and ingredients as percentage weight per weight (wt/wt %).

b Ice cream portion is 60 g; reduced-fat milk portion is 200 g.

c Whey powder (Avonol 600) was supplied by Glanbia Ingredients (Kilkenny, Ireland) and contained 55% (wt/wt) lactose, 31% protein, 2% milk fat, 0.69% calcium, and 7.3% other milk minerals.

d Milk minerals were CAPOLAC MM-525 supplied by Arla Food Ingredients (Viby, Denmark) and had a calcium content of 24% (wt/wt).

e Butterfat was supplied by Meadow Foods Ltd (Chester, UK) and contained 70% saturated fatty acids, mainly palmitic acid (C16:0, 31%) and oleic acid (C18:1n-9, 21%).

f Coconut oil was refined and supplied by Albion Chemical Distribution (Luton, UK) and contained 92% saturated fatty acids, mainly lauric acid (C12:0, 47%).

**Table 2:** Pre-study relevant baseline characteristics (n=16)^a^ of volunteers in the dual-label stable isotope calcium bioavailability study^b^

Characteristics	Value
Age (y)	35±7 (25-45)
BMI^c^	24±2.4 (19-32)
Serum 25(OH)D (ng/mL)^d^	35±6.4
Energy intake (kcal/d)	1,357±403 (739-1,995)
Protein intake (g/d)	69±5 (53-96)
Fat intake (g/d)	41±17 (13-64)
Carbohydrate intake (g/d)	166±68 (15-287)
Calcium intake (mg/d)	695±442 (203-1,726)

a All values are mean±standard deviation with ranges in parentheses.

b Nutrient values were calculated from 2 days food-intake diaries at baseline. There was no substantial change in dietary intake of nutrients or calcium during the study.

c BMI=body mass index; calculated as kg/m^2^.

d 25(OH)D=25-hydroxyvitamin D. To convert ng/mL 25(OH)D to nmol/L, multiply by 2.496.
